# Engineering Venom’s Toxin-Neutralizing Antibody Fragments and Its Therapeutic Potential

**DOI:** 10.3390/toxins6082541

**Published:** 2014-08-21

**Authors:** Larissa M. Alvarenga, Muhammad Zahid, Anne di Tommaso, Matthieu O. Juste, Nicolas Aubrey, Philippe Billiald, Julien Muzard

**Affiliations:** 1 Muséum National d'Histoire Naturelle, UMR MNHN-CNRS 7245, 12 rue Buffon, 75231 Paris Cedex 05, France; E-Mails: lmalvarenga@gmail.com (L.M.A.); zahid@mnhn.fr (M.Z.); 2 Université de Tours—INRA, UMR 1282, Faculté de Pharmacie, 31 Avenue Monge, 37200 Tours Cedex, France; E-Mails: anne.ditommaso@univ-tours.fr (A.T.); matthieu.juste@univ-tours.fr (M.O.J.); aubrey@univ-tours.fr (N.A.); 3 Université Paris-Sud, Faculté de Pharmacie, IPSIT, 5 rue J.-B. Clément, 92296 Châtenay-Malabry, France; 4 Molecular Foundry, Lawrence Berkeley National Laboratory, 1 Cyclotron Road, Berkeley, CA 94720, USA; 5 Department of Zoology Islamia College University, Peshawar 25000, Khyber Paktunkhwa, Pakistan

**Keywords:** scorpion stings, snakebite, antivenom, serum therapy, antibody, scFv, diabody, nanobody

## Abstract

Serum therapy remains the only specific treatment against envenoming, but anti-venoms are still prepared by fragmentation of polyclonal antibodies isolated from hyper-immunized horse serum. Most of these anti-venoms are considered to be efficient, but their production is tedious, and their use may be associated with adverse effects. Recombinant antibodies and smaller functional units are now emerging as credible alternatives and constitute a source of still unexploited biomolecules capable of neutralizing venoms. This review will be a walk through the technologies that have recently been applied leading to novel antibody formats with better properties in terms of homogeneity, specific activity and possible safety.

## 1. Introduction

Niels Bohr used to say “Predictions are very difficult especially concerning the future”, and one could add “the best way to protect yourself is to make diversity”. Venomous animals have understood this principle in a far better way that is well exemplified by their venom. Indeed, venomous animals produces venoms that are very rich in bioactive compounds, whose pharmacological activities enable them to capture and immobilize the preys on which they feed, but also defend themselves from predators and microorganisms. This is particularly well illustrated with the scorpion, which is among the most ancient terrestrial arthropods. In addition to complex venom, they have developed protection against other aggression, such as oxidative stress, radiations, microorganisms and others, by producing protective molecules, such as hemocyanin and defensins, that patrol in the hemolymph [1,2].

Generally in vertebrates and particularly in mammals, the immune system has developed as a defensive barrier against pathogens and non-self substances. Antibodies circulate in the body, recognizing any foreign agents and contribute in removing them. The immune system keeps the memory of this attack for several decades in order to respond even more effectively in a future exposure to the same foreign agent.

In 1890, Von Behring and Kitasato took advantage of these observations by showing that the passive transfer of antibodies from an immunized animal to a non-immune animal could give protection against bacterial toxins [3]. In the more than 120 years since this discovery, scientists have been striving to develop targeted therapies based on the use of antibodies [4,5].

First, serum therapy had been widely used for the treatment of infectious diseases until the development of antibiotics. Today, the use of serum therapy is restricted to a limited number of viral and toxin-mediated diseases for which there are no alternative therapeutic options [6]. Sometimes, hyper immune human serum immunoglobulins from pooled human donors are used. This is the case for the treatment of acute infections, emerging viral diseases and influenza pandemics, but the benefit is controversial, and robust treatment data are lacking [7]. Thus, polyclonal heterologous antibody fragments (horse, sheep and rabbit) continue to be used in a number of niches, such as rabies, digoxin toxicity and envenoming treatments. The preparation of these therapeutics has taken advantage of several technological developments made over time, but it has largely missed the recombinant therapeutic antibody revolution of the past decades, which allows one to create novel formats of antibody fragments, whose functional and structural properties can be adjusted *in vitro* for specific applications [8,9]. In this way, recombinant antibodies are emerging as a new class of drugs with extremely high potential in a wide range of therapeutic applications, primarily autoimmune/inflammatory, oncology and, to a lesser extent, neovascular, infectious, hemostasis and transplant rejection [10]. In 2012, among 15 top-selling drugs, six were antibody-based molecules, and global sales of antibody-based therapeutic products exceeded 50 billion U.S. dollars. Many other therapeutic antibodies are in the pipeline, with more than four hundred in various stages of clinical trials, and several biosimilars are already under evaluation [11]. In this context of fast development, it is surprising that the treatment of envenoming has not yet taken advantage of such improvements, even if a number of preclinical studies clearly demonstrated the potential of anti-toxin recombinant antibody fragments.

In this review, we focus on improvements over time in the production of conventional antivenoms and alternatives that could now be considered in light of recent progress. Our goal is to review the state of the field and to identify areas where these novel recombinant biomolecules represent opportunities with additional value in terms of efficacy and safety.

## 2. Historical Development of Serum Therapy

The prophylactic and therapeutic potentials of immune serum were discovered by von Behring and Kitasato (1890), who showed that the passive transfer of antibodies from the blood of infected animals could provide immunity to diphtheria. In recognition of this discovery, Von Behring was the first recipient of the Nobel Prize for Medicine and Physiology in 1901. Soon after, during the Christmas night of 1891, Emil von Behring took advantage of this discovery and successfully treated a child for the first time using an anti-diphtheria serum. Industrial production then began using sheep sera that were gradually substituted for equine. In cases where humans are the only hosts of the pathogen, human convalescent sera have also been successfully used. Following the same approach, Césaire Phisalix and Gabriel Bertrand (1894) at the national Museum of natural History in Paris (F) demonstrated the antitoxic activity of the blood of a guinea pig immunized against the heat-inactivated venom of *Vipera aspis*, while Albert Calmette (Pasteur Institute of Saigon) was able to obtain antisera capable of protecting against envenoming from cobra bites, thus demonstrating the advantage of serum therapy in the treatment of envenoming [4]. After these pioneering discoveries, serum therapy has been extensively used for the treatment of numerous infectious and toxin-mediated diseases. In the 1930s, the discovery of the therapeutic value of penicillin ushered in the age of antibiotics and made a break from the use of immune serum for the treatment of infectious diseases [12]. Antibiotics quickly appeared to be easier to manufacture, less toxic to patients and produced efficient treatments [13]. In addition, industrial processes have allowed the production of antibiotics as stable compounds with consistent activity, while the activity of immune sera depends on animal sources that exhibit great batch-to-batch variations. Finally, and perhaps most importantly, many new antibiotics have a broad spectrum of activities, so that they can be prescribed without a specific diagnosis, while the use of antibody therapy requires accurate identification of the pathogen or the toxin responsible for the disease. As a consequence, serum therapy was unable to compete with antibiotics, and the therapeutic use of hyper immune animal serum has now been limited to the treatment of envenoming and rare diseases for which there are no alternative therapeutic options.

## 3. Conventional Anti-Venoms: Strengths and Weaknesses

### 3.1. Main Points

**Figure 1:**
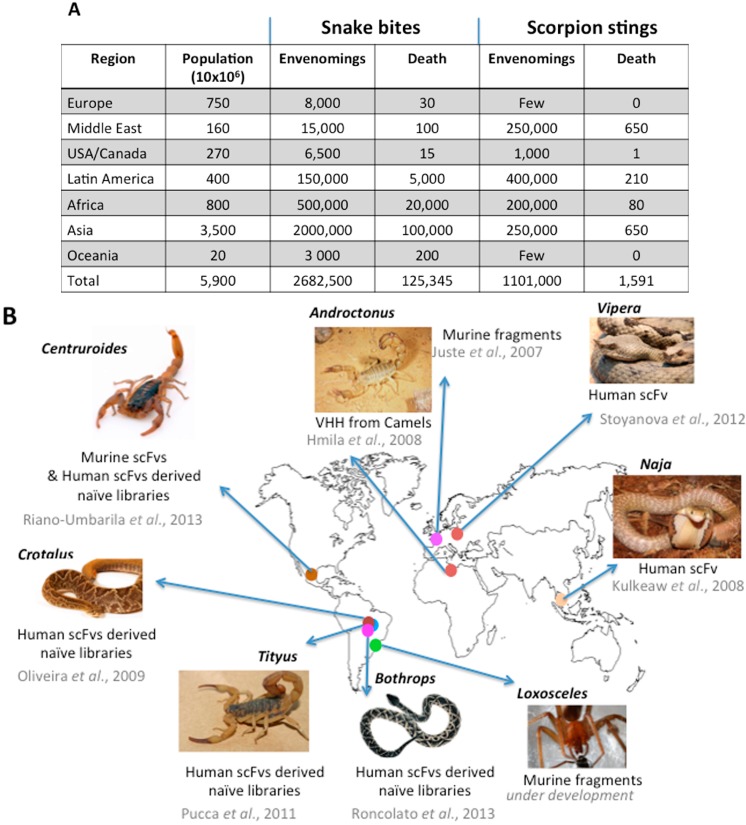
Snake bites and scorpion stings around the world. (**A**) World incidence and mortality adapted from [14,15]); (**B**) research carried out on recombinant antibody fragments with the potential for neutralizing venoms.

Envenomings are common emergencies in many countries across the world (Figure 1).

**Figure 2:**
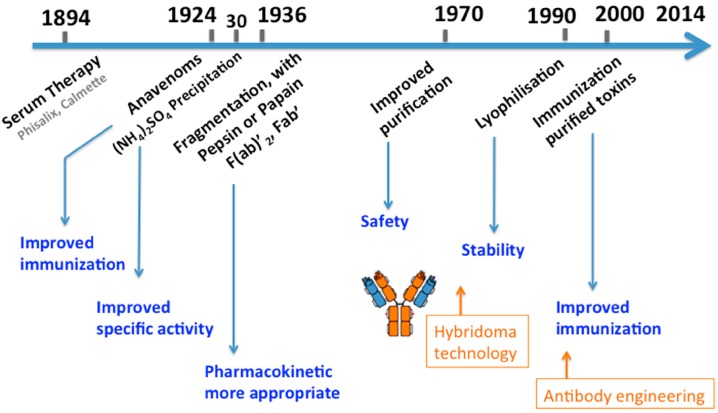
A timeline of polyclonal antivenom development. Antivenom production has taken advantage of several technological improvements in antibody fragmentation, protein purification and preservation, but no breakthrough innovation has yet taken place despite the discovery of hybridoma and antibody engineering technologies. Adapted from [20].

Nowadays, conventional serum therapy remains the only specific treatment of envenoming whatever the origin is: snake, scorpions, spiders or marine animals [14,16,17,18]. It involves the application of antibody fragments to inactivate and accelerate the clearance of relevant venom components from the victim’s system. Over time, the manufacturing of antivenoms has benefited from progress in immunology and analytical biochemistry, resulting in products with more appropriate pharmacokinetic (PK) and pharmacodynamic (PD) properties, improved specific activity and reduced risks of side effects, including life-threatening anaphylactic reaction (Figure 2) [19]. In other words, the main principle remains the same, and conventional antivenoms still consist of heterogeneous preparations made of many types of antibodies, out of which, only a minute fraction is specific for the potent toxins. Here, we will focus on the various points to be considered for the preparation of antivenoms.

### 3.2. Immunogens and Host Animals

**Figure 3:**
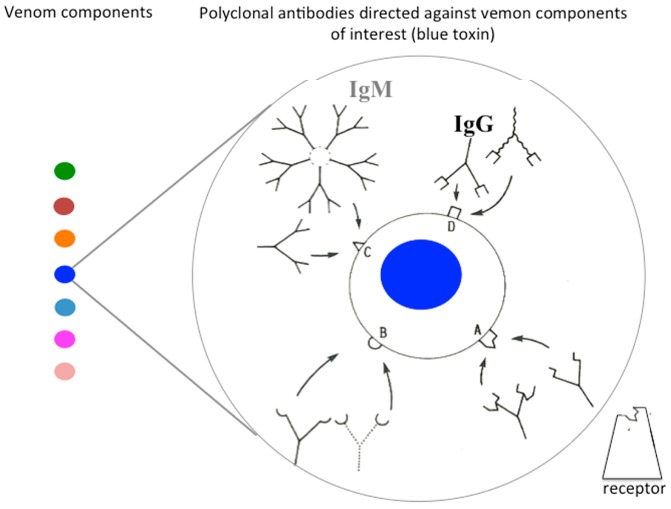
Recognition of a venom component by polyclonal antibody. Antibodies of interest are those that inhibit the interaction of the most potent toxins (blue) with their receptor. Such neutralizing antibodies bind to an epitope (A), which overlaps with the active site of the toxin, create steric hindrance or induce deleterious conformational changes in the toxin upon binding. Immunoglobulins (IgG, IgM) with distinct avidity can compete for the same epitope. Polyclonal antivenoms suffer from several drawbacks, such as heterogeneity and variability in their composition and efficacy, and may be associated with side effects, such as tolerance, when injected into humans.

The very first points to be considered are the choice of immunogen to be injected into the host animals and the schedule of immunization. Most of the time, antivenoms are prepared after repeated injections of whole venom or toxic fraction to host animals. Horses are the most commonly used animals, due to the great volume of blood that can be collected, but their serum contains several subclasses of IgG, including highly glycosylated IgGT with a protective ability, but powerfully immunogenic when injected into other species, especially to humans [21]. Sheep are safer, because they lack IgGT, but produce serum in a lower amount. One also must remember the use of polyclonal rabbit F(ab')_2_, as in the case for *Atrax* antivenoms in Australia. Serum is usually collected after bleeding of animals, but plasmapheresis may be preferred with the advantage of keeping the host animal alive. The nature of the immunogen injected into animals is also to be taken into account. The vast majority of producers usually use healthy horses that are immunized with crude venom and promote immune responses with complete and incomplete Freund’s adjuvant. Such protocols may last for a long time (9–12 months). However, no more than 20% of the animal antibodies will be directed against the venomous components, of which, less than 5% are neutralizing antibodies, some of them being capable of targeting overlapping neutralizing epitopes, but with different affinities (Figure 3). This contributes to a low activity per mass of protein, also designated specific activity, and frequently leads to the administration of life-threatening volumes that compromise the safety and effectiveness of the treatment [22].

Reported alternatives are the use of partially detoxified venoms, still capable of eliciting the production of neutralizing antibodies, or venom extracts partially fortified in the most potent toxins. The use of a limited number of recombinant toxins has also been suggested, but still awaits development [23].

### 3.3. Fragmentation and Purification Process

Usually, antibody fragments obtained by limited proteolysis are preferred as compared to whole immunoglobulins, due to the better PK profile compared to whole IgGs [24]. Most producers have adopted similar methodologies to manufacture antivenoms. These consist of limited digestion with pepsin or papain in order to get F(ab')_2_ or Fab fragments, followed by salt or caprylic acid precipitation. Additional steps include ion-exchange chromatography to remove traces of contaminants, including albumin, which is one of the most immunogenic components of the serum. Bacterial and viral safety steps, like pasteurization, are also undertaken, so that the risk of infectious disease transmission remains theoretical [25].

Fragmentation of IgGs contributes to a decrease of the risk of a hypersensitivity reaction by removing the Fc fragment of IgGs. However, papainolysis is difficult to standardize, and some reports consider that Fabs are less effective than F(ab')_2_, both in their distribution and neutralization activities [26]. Conversely, sometimes, pepsinolysis leads to the alteration of the antibody function or aggregation, due to exposure to the harsh low pH used (pH 2.0) and up to a 20% destruction of the antigen-binding site.

Furthermore, batch-to-batch variation in enzyme quality and pH can result in inadequate digestion, loss of activity and poor quality F(ab')_2_ preparation with a high incidence of early adverse reactions when injected into humans.

All of these steps have been considered in protein purification approaches over the past century, but still need amelioration. Indeed, the processes usually do not include any affinity purification of antibodies against the toxic components of the venom. This point is essential, since it contributes to the limited specific activity of antivenoms, which contain antibody fragments with all sorts of antigen-binding activity. The risk of adverse reactions is increased, due to a higher amount of total protein injected, Fabs with unrelated activity and non-neutralizing antibodies that can compete with toxin neutralizing antibodies, due to steric hindrance [25].

Considering all of these points, polyclonal preparations remain not chemically well-defined reagents and exhibit high lot-to-lot variability, differing over time and by source of origin [27]. All of these antibodies are from a heterologous origin. No human polyclonal antibodies, as is the case for anti-D antibodies or anti-infectious diseases, such as rabies and tetanus, or human polyvalent immunoglobulin IgIV can be prepared, because immunization of humans with venoms or anatoxins is not conceivable [28]. Thus incidence of early adverse reaction remains high, ranging from 5% to 80%, depending on the quality of the preparation [15,22].

## 4. Monoclonal Antibodies: Precursor of Therapeutic Antibodies

In the 1970s, Milstein and Kohler’s work opened the way for the production of monoclonal antibodies for diagnostic and therapeutic purposes [29]. This technique allowed the selection of antibodies, also designated monoclonal antibodies, because it refers to a population of antibody molecules that contains only one species of an antigen-binding site capable of immunoreacting with a particular epitope. The technology consists of creating hybrid cell lines (hybridoma) by fusing a specific antibody-secreting B-cell with a non-secreting antibody myeloma cell, selected for its ability to grow in tissue culture. The resulting hybridoma cell is immortal and will produce forever the same antibody with a defined specificity and a single isotype, the so-called “magic-bullet” [30].

In this way, the hybridoma technology meets the challenge that 100% IgGs of an antivenom are therapeutic, provided that their selection is carried out on the neutralizing capacity of the secreted antibody. This discovery has raised many hopes and enthusiasm for the production and development of innovative drugs having additional advantages in terms of reproducibility and specific activity in contrast to polyclonal preparations [31].

However, the production of human monoclonal antibodies has mainly been a resounding failure, and only monoclonal antibodies from rodent origins are commonly produced against any antigens. The constant domains of these rodent antibodies are more immunogenic than others when injected into humans [32]. In addition, their immune effector functions, such as antibody-dependent cellular toxicity (ADCC) and complement-dependent cytotoxicity (CDC), are not adapted to humans. As a consequence, rodent antibodies have hardly contributed anything in medicine and are generally not opted for with respect to therapeutic purposes. It is evident that only three have been approved by the FDA for clinical applications. Concerning venoms, many mouse monoclonal antibodies have been generated against animal toxins. Some of these antibodies are neutralizing, capable of increasing survival time in animal experiments and even to reverse toxin binding to their receptors [33,34,35]. These monoclonal antibodies cannot be considered as potential drugs for the reasons mentioned above, but they provide appropriate templates to engineer recombinant therapeutic molecules suitable for human administration [36].

One must also notice that transgenic mice expressing human Ig genes provide an alternative method, but this technology has been patented and never used for isolating anti-toxin human antibodies [37].

## 5. Recombinant Antibodies and Antibody Fragments: New Opportunities

*In vitro* molecular engineering methods have seen impressive advances in recent decades, making possible the design of recombinant therapeutic antibodies. Thus, it has been possible to make monoclonal antibodies more human and less immunogenic, to adapt their PK/PD properties to particular applications by creating new formats of fragments with an increased plasma half-life and high tissue-penetration capacity, to modulate their functional properties in terms of affinity and specificity for their target by mutagenesis and, finally, to alter their biophysical and biochemical characteristics contributing to higher solubility and improved stability [9].

### 5.1. Making Monoclonal Antibodies More Human

**Figure 4:**
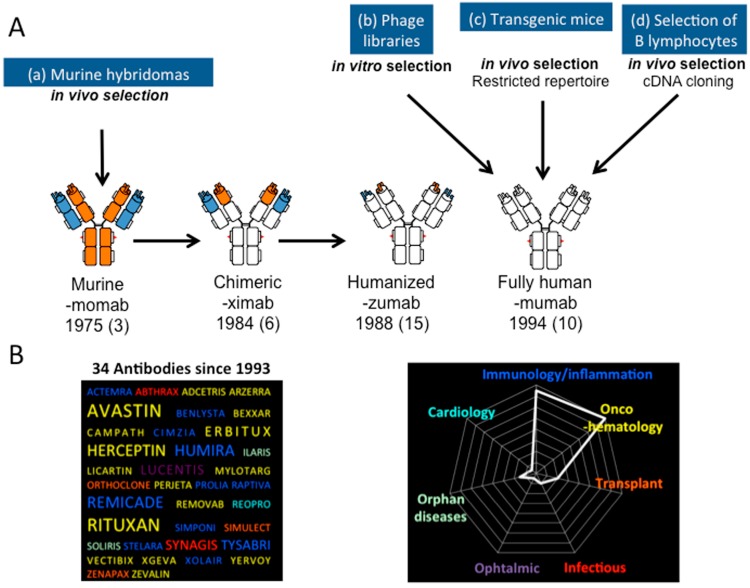
The design of therapeutic antibodies. (**A**) Therapeutic antibodies can be produced after re-engineering of murine monoclonal antibodies, whose domains are represented in blue and orange (**a**); leading to chimeric or humanized antibodies that are less immunogenic when injected into humans, while preserving antigen-binding properties (antibody domains from human origin are represented in white). Alternatively, *in vitro* panning of phage libraries displaying antibody fragments followed by grafting onto human antibody constant domains allows *in vitro* selection of fully-human antibodies (**b**); *In vivo* selection of fully human antibodies can also be performed by using transgenic mice (**c**) or single peripheral blood B-cells and cDNA cloning (**d**); Various types of engineered full-sized antibodies are indicated here with the suffix used in the international non-proprietary name (INN), the first year of marketing and the number of molecules approved up to now for the treatment of various diseases, but not envenoming (in brackets); (**B**) Therapeutic antibodies now represent the fastest growing class of biodrugs approved for therapeutic treatments. (**Left**) The best-selling molecules are represented with the word size proportional to their respective market share. (**Right**) The distribution of all therapeutic antibodies according to their indication.

Different strategies have been implemented to make a murine antibody less immunogenic in order to reduce the risk of human anti-mouse antibody (HAMA) response when injected into humans (Figure 4).

Initially, the murine constant domains of the antibody have been substituted for their human counterpart, resulting in chimeric human/mouse molecules that preserve the original antigen-binding functions and characteristics, but lack more than 60% of the murine parental sequence and, thus, are expected to be less immunogenic. Nowadays, six out of 34 antibodies approved by the FDA are chimeric. Humanization of antibodies is another approach, which was developed later, leading to antibodies with even greater similarities, but is more tricky to be implemented [38]. It consists of grafting murine complementarity-determining residues (CDRs) onto antibody frameworks of human origin, leading to molecules whose sequences are extremely similar to human antibodies. However, this approach often alters antigen-binding properties, since the nature of the frameworks greatly affects the proper folding of the CDRs. This is the reason why alternatives to CDR-grafting have been considered and allowed further progress: resurfacing simply consists of the comparison of the variable domains with those of human antibodies whose three-dimensional structure has been solved and then exchanging the solvent-exposed residues. It is very simple, because a limited number of mutations are considered, and this usually allows one to retain the antigen-binding properties. Other rational methods have been described, such as super-humanization or human string content optimization, but often showing reduced affinity or even loss of affinity. Besides, a number of non-rational methods based on generating large combinatorial libraries and the selection of variants upon antigen-binding activity have been reported. These methods include framework libraries, guided selection and framework shuffling.

More recently, it has also been possible to obtain fully-human monoclonal antibodies from a natural recombinant repertoire, especially after generating large combinatorial libraries, and *in vitro* selection of antibody fragments with the desired specificity by enrichment technologies, such as phage, ribosome or yeast display, or by high throughput screening techniques [39,40]. These methods are powerful new tools for making antibodies outside of the immune system, thus avoiding animal immunization [41]. Among them, scFv-phage library panning has become the most popular. The main advantage of this technology is the link between phenotype and genotype, since each phage carries in its genome the DNA encoding the antigen-binding protein displayed on its surface. However, sometimes it happens that the antibody fragment that appears to be an excellent ligand when fused to the phage-protein is difficult to express as free scFv, lacks stability and shows poor antigen-binding [42].

Alternatively, human monoclonal antibodies can be prepared from transgenic mice, which in theory includes an intrinsic selection for well-expressed, non-aggregating antibodies. Even more recently, a technique was reported for isolating monoclonal antibodies from human peripheral blood mononuclear cells, associated with antibody V-domain amplification, cloning and expression [43]. All of these clearly demonstrate the intensity of research over the past 20 years in this highly competitive field and the potential of antibodies to be designed as therapeutic molecules.

### 5.2. Enlarging the Diversity of Antibody Fragment Formats

One major disadvantage of antibody fragmentation using conventional enzymatic methods is the limited number of functional fragments that is restricted to Fabs and F(ab')_2_ and sometimes associated with aggregation and loss of activity.

**Figure 5:**
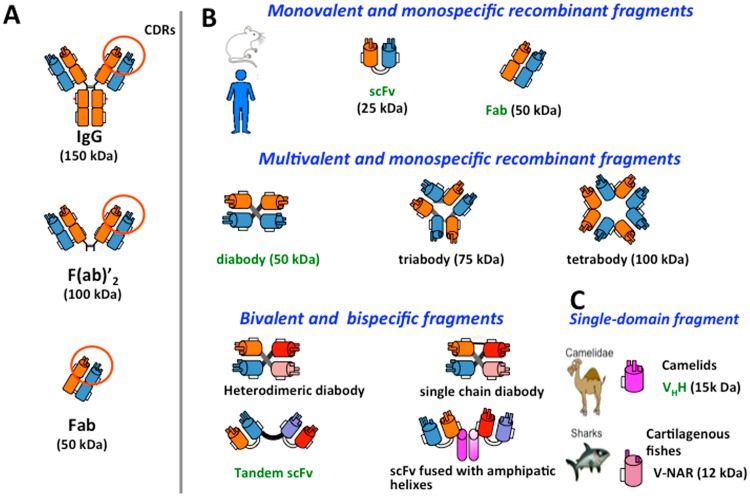
Tailoring antibody fragments. (**A**) Limited proteolysis of whole IgG molecules allows one to prepare functional F(ab')_2_ or Fab fragments; (**B**) molecular engineering allows one to design new formats of antibody fragments, mono- or multi-valent, mono- or bi-specific. These molecules are essentially derived from monoclonal antibodies or antibody phage libraries. They differ from each other in size, PK and PD properties when injected into humans or animal; (**C**) Recombinant antibody fragments derived from camelids or cartilaginous fish constitute alternatives to conventional antibodies, because their antigen-binding site simply consists of a single antibody domain of 12–15 kDa. In green are indicated some of these molecules under development in research or in the early preclinical phase for the treatment of envenomings.

Through versatile techniques of genetic engineering, genes encoding the antibody V-domains can be cloned and antibody domains combined in a variety of ways to form antigen-binding molecules with unprecedented forms (Figure 5) [44]. These molecules can be produced in host cells (bacteria, yeast, insect and mammalian) [45]. These new formats can be used, for instance, to improve diffusion into tissues, to increase the bioavailability of the antibodies and also to make them less immunogenic [46]. In this way, there is a nascent shift toward the study of antibody fragments in clinical development [8]. The minimal size of a functional antibody fragment is the scFv, in which the variable domains of each chain (VH and VL) are associated with each other via a short flexible peptide that maintains the contact and allows proper folding. Fifteen residue linker ((G_4_S)_3_) have been reported to induce the formation of monovalent, monomeric molecules [47]. Shorter linkers (five residues) create a constraint between the variable domains of the same molecule, which favors the formation of a dimer, also called a diabody, which has the same size as a Fab fragment (50 kDa), but is bivalent [48]. An even shorter linker (one residue or even no residue) results in trimeric (triabody) or even tetrameric (tetrabody) multivalent assemblies with higher avidity for their target. Analysis of many diabodies has shown a better retention in targeted tissues and a lower systemic clearance [46]. Several of these molecules are now in active clinical development and many others in the preclinical pipeline [8].

Molecular engineering has also given the possibility to create bispecific antibody fragments that simply consist of variable domains of two different antibodies. Several arrangements are possible: a heterodimeric diabody, in which one subunit is made of the VH of the first antibody fused to the VL domain of the second one with different antigen-binding specificity. The second subunit consists of the VH of the second antibody fused to the VL of the first one. Such a dimeric structure is very compact and rigid. An alternative is the tandem-scFv in which two scFvs directed against different targets are associated via a short peptide linker. Tandem-scFvs are even more flexible. Other techniques rely on molecular domains that have a propensity for self-assembly. As an example, scFv can be produced by fusion with amphipathic α-helix, “coil-coil” structures or antibody constant domains, leading to multivalent molecules [49].

Finally, functional single-domain recombinant antibodies (sdAb), also called nanobodies, have been reported [50]. At the early stages, these molecules were derived from common human or murine Ig by splitting the variable domains [51]. However, these molecules had poor solubility, reduced affinity for their target and irreproducible outcomes. Later on, single-chain antibodies, also called HCabs, for their unique functional heavy (H)-chain antibodies, have been reported in camelids (dromedaries, camels, llamas and alpacas). The H chain of these homodimeric antibodies consists of one antigen-binding domain (VHH) and two constant domains. The functional unit (VHH) has a low MW (15 kDa) and contains three CDRs [52]. Cartilaginous fish, such as sharks, also produce immunoglobulin-like proteins designated IgNAR, which consist of homodimers with no associated light chain [53]. The functional unit of these molecules is a single domain (VNAR) of 13–14 kDa with only two CDRs. All of these single-domain antibodies could combine the benefits of conventional antibodies in terms of selectivity and affinity with important features of small molecule drugs, such as a higher tissue penetration rate. However, their fine PK/PD properties and kidney clearance remain to be investigated in detail.

## 6. Preclinical Evaluation of Recombinant Antibodies for the Treatment of Envenomings

While serum therapy is widely used for the treatment of envenomings regardless of their type of envenomation (snakes, arachnids), the research in the field of recombinant therapeutic antibody fragments varies greatly depending on the type of envenoming. In fact, the interest in recombinant antibody technology will depend on the nature of the venom. Here, we focus on the most recent advances in the design of recombinant antibodies against snake and scorpion toxins. We also know that occasional studies have been undertaken for developing antidotes against other venoms [54].

### 6.1. Snake Venoms

Usually, antivenoms are appropriated to the treatment of snakebite, even if their clinical efficacy remains to be improved [55]. In this way, several developments are under investigation, especially in the process of the production of conventional antivenoms to make them safer and more efficient [56]. However, there are few studies concerning the development of recombinant antibody strategies, and this may be related to the complexity and unique mixtures of biologically active compounds that affect different vital systems of the victims [57]. Recombinant antibodies are raised against a single epitope and, unlike polyclonal antivenoms, are not well-suited to neutralize complex mixtures in which the major toxic effects are not due to one, but several toxins with different immunogenic profiles and various biological activities, such as hemorrhage, myotoxicity and neurotoxicity [58,59,60]. Complete neutralization of snake venoms would require one to generate a pool of toxin-specific recombinant antibodies or to use them as additive components of polyclonal preparations. In the latter case, it would certainly be preferable to use conventional antivenoms prepared after immunization of animals against a cocktail of the most potent toxins instead of the whole venom [61]. An interesting alternative would be to design epitope-string immunogens using bioinformatics to generate IgGs against the most clinically relevant toxins and to take into account the variation in the representation and toxicity of venom components. In addition to horses and sheep, camelids may be considered as a potential host to generate antivenoms [62,63]. The unique structure of HCabs may account for the report that camel antibodies should be less immunogenic, less likely to activate a complement and potentially safer. Despite these considerations, several studies have focused on the engineering of recombinant neutralizing antibody fragments directed against snake venoms [64,65,66,67]. Most consisted of the panning of human naive scFv-phage libraries (Griffin. 1, MRC Cambridge, UK; Tomlinson I + J, Cambridge, UK) and allowed to isolate scFvs, but all with poor neutralizing capacity. In the best case scenario, only partial inhibition of venom’s activities were observed *in vivo*, leading to the conclusion that these molecules are simply candidates for inclusion in a mixture of specific antibody fragments to produce antivenoms. This point could be related to the high diversity of toxins in the venom that cannot be simultaneously neutralized by an antibody fragment specific for a single epitope, but also to the intrinsic functional properties of the scFv selected from naive libraries that have not undergone stringent maturation processes and therefore exhibit low affinity for their target. Recently, a study on the panning of a humanized-camel VHH-phage display library led to the selection of single domain antibodies; here again, with mixed results in terms of neutralizing capacity [68].

### 6.2. Arachnid Venoms

In the case of arachnids, envenomings are simpler. The diversity of lethal toxins in scorpion venoms is much lower than in snakes, and their molecular and pathogenic identities are now better understood [15]. Neurotoxins are the main components of medical importance, due to their high toxicity for humans and relative abundance. These toxins bind to specific ion channels (Na_v_ and K_av_) with affinities in the nanomolar range. Na_v_-specific toxins are mainly responsible for the lethality, at least in animal models. They cause direct impairment of Na^+^ channel activity, the function of the peripheral and central nervous system and disrupt the transmission of nerve impulses, leading to vital physiological defects.

Toxins acting on voltage-gated sodium channels of excitable cells are low MW polypeptides (6.5–7 kDa) with a well-conserved tertiary structure, acting on Sites 3 or 4 of Nav. The nature of these toxins may greatly vary from one scorpion species to the other, but within each species dangerous for humans, the lethality of the venom is caused by a very limited number of toxins. Depending on the scorpion species, they belong to one or no more than a couple of immunogenic groups [69]. Thus, it becomes possible to neutralize the main effects of venoms simply by preventing the binding of these toxins to Na channel cell receptors by using one or two monoclonal antibodies.

All scorpions dangerous for humans belong to the Buthidae, and those capable of inflicting fatal stings occur in Central and South America (*Tityus* and *Centruroides*), North Africa and the Middle East (*Androctonu*s, *Buthus*, *Leiurus*), South Africa (*Parabuthus*) and India (*Mesobuthus*). However, research on neutralizing recombinant antibody fragments has been limited to venoms belonging to the American genera, *Tityus* and *Centruroides*, and to the North African species, *Androctonus australis*.

#### 6.2.1. *Tityus*

Two research groups independently isolated scFvs that recognize toxins from the *Tityus serrulatus* venom, and in both cases, this was carried out after panning human naive libraries [70,71,72]. By opting for this strategy, researchers avoided long-term animal immunization protocols and focused on the expected lack of immunogenicity of human-derived molecules. In order, to select toxin-binding antibody fragments, they probably carried out the panning of the libraries under conditions that were not stringent enough. The authors also faced some difficulties in producing free recombinant antibody fragments initially expressed as good binder phage-antibodies, and finally, they did not analyze the antigen-binding constants. As a consequence, the scFvs they reported showed low potential for future therapeutic applications. ScFv 2A (also designated Serrumab) was demonstrated to inhibit cytokine production in cell lines exposed to the venom. It also slightly reduced the alteration of biochemical (plasma levels of urea, creatinine, AST and glucose) and immunochemical parameters in mice receiving venom pre-incubated with Serrumab. However, none of the effects were in any case better than those observed with a commercial scorpion antivenom (Butantan Institute) consisting of whole conventional polyclonal IgGs, even if used at a much lower molar concentration as compared to the scFv preparation. Amaro *et al.* reported the selection of another human scFv directed against Ts1 toxin capable of cross-reacting with the venom of other species from the same genus [70]. *In vivo* assays allowed them to demonstrate the neutralizing capacity of this scFv in mice, but only challenged with 1 LD_50_ of Ts1 pre-incubated with a large excess of scFv (molar ratio toxin:scFv: 1:10). Once again, the weakness in the results is mainly due to the low antigen-binding affinity of such antibody fragments derived from naive libraries that have not undergone sophisticated maturation processes, as is the case in the *in vivo* immune response. All together, these observations did not make it possible to consider *in vivo* assays as life saving.

#### 6.2.2. Centruroides

**Figure 6:**
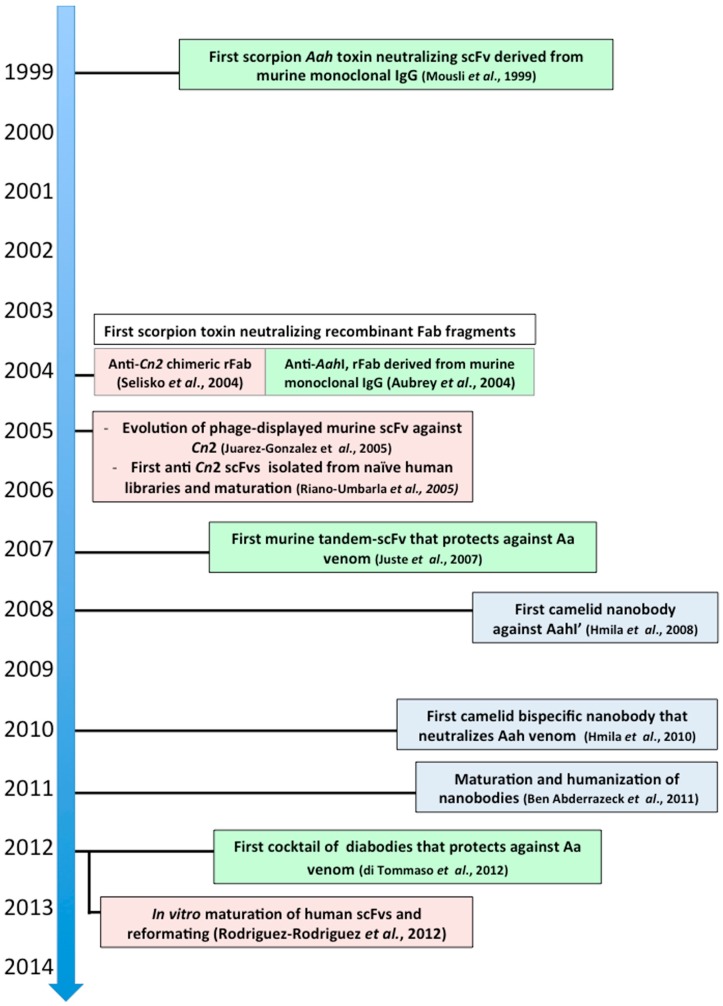
Third generation of neutralizing scorpion toxin antibody fragments. Here are indicated the most significant and pioneering steps in the process of designing new therapeutic candidates. These molecules have been genetically engineered and designed for improved protective capacity against experimental envenomings. Recombinant antibody fragments from murine (green), human (pink) or camelid (light blue) origins.

Studies undertaken to design recombinant antibodies with the potential for the treatment of *Centruroides* stings bring the utmost significant advances, because of several improvements and considerable efforts from the Mexican group, Universidad Nacional Autonoma de Mexico, Cuernavaca [73]. Here, the first point of significance is the panning of naive libraries against purified toxins and not the whole venom (Figure 6). This strategy allowed for the restriction of the selection of antibody fragments to well-identified components that play a key role in the envenoming process. Initially, the authors showed the poor *in vitro* toxin-neutralizing capacity of the human scFvs (3F and C1) that they isolated from naive libraries, either because these scFvs not being target appropriate epitopes or due to their low affinity in the range of 10^−7^ M [74]. In an interesting and original way, the authors submitted these scFvs to *in vitro* maturation processes in a completely non-rational engineering way [75]. This strategy involved error-prone-PCRs under conditions to obtain low random mutations rates (0.5%–2%) followed by the expression of random mutants as scFv-phage libraries and bio-panning rounds against scorpion toxins. Finally, several phage-scFv variants were selected and then evaluated as soluble scFv proteins. By using this strategy and three rounds of panning against *Centruroides noxius* (Cn2) toxin, the authors were able to convert scFv 3F into scFv 6009F, whose affinity for Cn2 toxin was improved by 446-fold (410 pM) and allowed to neutralize 2 LD_50_ of Cn2 toxin when injected into mice after being pre-incubated with the toxin in a 1/10 molar ratio. ScFv 6009F differed from scFv3F by only six residues of which, three are located outside of the CDRs. These results indicate that improving neutralizing capacity may not only depend on residues directly involved in the interaction with the targeted epitope, but also on residues of the antibody frameworks that contribute to the stability of the recombinant molecule.

Another advantage of the *in vitro* maturation process is the possibility given to modulating the antigen-binding specificity. Following the strategy reported above and using the same scFv3F as a starting point, but now by panning the library against the closely related toxin Css2 from *Centruroides suffusus suffusus*, an scFv variant, designated 9004G, which preserved high binding-affinity for Cn2 while being able to cross-react with Css2, was selected. scFv 9004G neutralized 2 LD_50_ when injected into mice after pre-incubation with each toxin (Cn2 or Css2) in a 1/10 molar ratio. As compared to scFv3F, six point mutations were found to be fundamental to generating this cross-reactivity, but surprisingly, none of them was located in the CDRs, which contain the residues expected to interact with the epitope. Such mutations may contribute to increasing the stability of the scFv. Structural studies and X-ray analysis of the 9004G-Cn2 complex interface confirmed that epitope 9004G mainly involves residues of Cn2 that are common with Css2 toxin and located in the pharmacological site of both toxins.

Finally, the same group took advantage of both independent maturation processes reported above. A variant of 9004G in which residue V 101 in CDR H3 was substituted for F, as the case is in scFv 6009F, was created by site-directed mutagenesis. The resulting scFv (scFv LR) exhibited improved functional properties and neutralized 2 LD_50_ of Cn2 toxin when injected into mice after being pre-incubated with the toxin in a 1/2 molar ratio [75]. This output must be considered as a remarkable achievement (Figure 6). Indeed, beneficial point mutations generated from independent maturation processes usually appear to be neutral or deleterious when combined [76]. A final variant (scFv LER) with increased thermal stability was created after a single point mutation of scFv LR in Framework 2 of the heavy chain capable of neutralizing 2 LD_50_ of Cn2 toxin with no detectable symptoms when injected into mice after pre-incubation with the toxin in a 1/1 molar ratio [77].

All together, these studies can be considered as the most successful when starting from fully-human scFv-phage naive libraries. However, this strategy requires a long process of molecular engineering in addition to the initial screening of the original naive library in order to select mutants with the expected specificity, affinity, stability and neutralizing capacity. *In vitro* mimicking of the natural immune system appears to be tricky when designing neutralizing antibodies directed against scorpion toxins. Improving functional and structural properties of these scFv have required a long step-by-step blind mutation process, which occurred not only in CDRs, but also in the framework with the potential risk of the original molecule affecting humans and making it potentially more immunogenic in the case of injection into humans.

Beside these analyses, which focused on affinity maturation and specificity extension, reformatting of the selected scFv was also investigated, but in much less detail. This point is crucial, since the protective capacity of an antivenom highly depends on its tissue distribution and penetration and also its capacity to redistribute the toxin in the envenomed organism [24]. It is usually considered that the plasmatic half-life of scFv is very short with a rapid distribution into tissues, but also elimination via kidney filtration and, thus, not well-suited for therapeutic applications [78]. Fabs are the utmost appropriate, and their properties meet the balance between rapid distribution and prolonged half-life. Their volume of distribution in the envenomed organism is 4–5-times higher than the plasma volume, and they can be cleared in complex with Na-channel toxins more rapidly than whole IgGs or F(ab')_2_, because their MW is below the threshold of renal filtration. Diabodies would be even more advantageous, because they combine both pharmacokinetic properties, similar to Fabs and bivalency. When investigating 6009F expressed in different formats (scFv, diabody and scFv), the authors concluded that the Fab format was superior to scFv or diabody. However, the assays were performed by pre-incubating the toxins with antibody fragments before being injected into animals, thus ignoring the pharmacokinetic properties of free molecules, as is the case in natural envenomings and experimental rescue assays [79]. The results so far reported are likely to reflect a loss of affinity of the diabody in relation to non-optimal design and do not allow one to draw general conclusions.

#### 6.2.3. *Androctonus australis*

The most dangerous toxins of the venom of the North African scorpion, *A. australis*, are sodium channel toxins that bind to Site 3 of the channel and inhibit the inactivation phase of the potential of action. Almost all of the toxicity of the venom accounts for three of these toxins, which present less than 5% of the total protein fraction of the venom. These toxins belong to two distinct structural and immunological groups [69]. Group I consist of toxins AahI and AahIII, whose sequences are closely related (78% identity), while Group II simply consist of toxin AahII, which is considered as the most potent animal neurotoxin. The LD_50_ for AahII in C57BL/6 mice weighing 20 g is 180 ng for sc injection *versus* 1 ng for icv injection. Neutralizing the whole venom should at least require a cocktail of two monoclonal antibody fragments with distinct specificity capable of neutralizing both groups.

The potential of recombinant antibody technologies to design scorpion toxin neutralizing-antibody fragments was first demonstrated by Mousli *et al.* with a strategy quite different from the one reported for *Tityus* and *Centruroides* scorpions [80]. It comprised the use of well-characterized hybridoma cell lines secreting neutralizing immunoglobulins that were affinity B-cell matured. The main advantage of this route is the quality and specificity of the resulting probe. In addition, the protocol is easily accessible for research groups who may not be specialized in this area and allows one to create recombinant antigen-binding molecules within less than 50 working days [44]. Monoclonal antibody 9C2 and 4C1 directed against toxins AahI/AahIII (immunological Group I) and AahII (immunological Group II) were reformatted into scFv [80,81]. Neutralizing capacities of the antibody fragments were investigated after intracerebroventricular injection of the antibody fragment pre-incubated with toxins. All of the scFv fragments directed against *Androctonus* toxins generated in this way were found to retain the functional properties of the parental antibody in terms of antigen-binding specificity, affinity and *in vitro* neutralizing capacity [80,81].

To identify the antibody format most suitable for *in vivo* protection, scFv 9C2 was redesigned into recombinant Fab and diabody fragments with an intermediate size [82,83]. The neutralizing capacity of the antibody fragments was investigated after intra-peritoneal injection of the antibody fragment pre-incubated with AahI. Diabody 9C2 was shown to be capable of neutralizing 4 LD_50_ of AahI toxin when injected into mice in the ratio of the toxin/antibody fragment of 1/2. Similar results were observed with Fab obtained via papainolysis, but corresponding to a higher mass of protein injected, while the recombinant Fab exhibited poor neutralizing capacity. The later result was a consequence of low active concentration or recombinant Fab related to partial non-proper folding and lack of stability [83]. Conversely, diabody 9C2 was shown to be fully bifunctional with a high apparent affinity for toxin AahI (K_D_: 8 × 10^−11^ M) similar to the parental murine IgG and without requiring any *in vitro* maturation process. In addition, diabody 9C2 exhibited high thermal stability in serum.

In the late 1990s, injection by the icv route of antibody fragments pre-mixed with toxins was considered as the gold standard to investigate the neutralizing potential of recombinant antibody fragments, because it required low amounts of product. Such an assay allowed one to demonstrate the capacity of scFv 4C1 (or scFv 9C2) to neutralize toxin AahII (or AahI) in the same way as the parental antibody. Nevertheless, such assays did not allow one to assess the PK properties of the antibody fragment, a parameter that is essential for successful therapeutic application and protection. The injection of the antibody fragments by a route different from the one of the toxin (ip/iv against sc) and a delay between the injection of the toxin or the venom and the treatment allow a better evaluation of the properties of the antibody or its fragments. The first assay of protection using recombinant antibody fragments was reported soon after and clearly demonstrated the significant advantages of larger recombinant antibody fragments, such as diabodies [82]. Diabody showed an excellent protective capacity in conditions that mimic clinical situations. All mice experimentally envenomed with 2 LD_50_ of AahI injected by the subcutaneous route survived when treated 10 min later by the intraperitoneal injection of diabody 9C2 in a 1/8 toxin/antibody ratio. Later, such assays were carried out with nanobodies, but led to mixed results [84].

Protection against the whole venom of Aa requires targeting toxins that belong to two distinct immunological groups. For this purpose, a tandem-scFv comprising two scFvs (4C1 and 9C2) fused together via a short peptide linker was designed. Here, again, the tandem-scFv retained high affinity for the targeted toxins with a K_D_ in the same range as the parental antibodies, reflecting the stability of the immune-complexes. This point is essential for the stable inactivation of the venom, to reverse the normal distribution of toxins in the body and to favor their clearance. Peritoneal injection of tandem-scFv was found to protect mice experimentally envenomed by the subcutaneous route, and the estimated protective capacity of the tandem-scFv was higher than 100 LD_50_ of venom per mg of tandem-scFv. This result counts as a remarkable achievement, since the neutralizing capacity of conventional antivenoms determined under extremely favorable conditions where venom and antivenom are preincubated together before being injected ranges between 0.5 and 5 LD_50_ per milligram of antivenom. A further step was taken when our group evaluated the protective capacity of a diabody mixture in which the molar ratio matches the characteristics of toxins and the polymorphism of the venom. The mixture consists of the diabody 9C2 (anti-group I) and diabody 4C1op (anti-AahII), the latter being modified to facilitate *in vitro* production and purification. The effectiveness of the antivenom was tested *in vivo* under conditions simulating scorpion envenoming. Here, again, the protective capacity observed was in the range of 100 LD_50_ per mg of diabody [85]. Advantageously, this strategy makes it possible to administer two different antibody fragments in a quantity adjusted according to various important factors, such as the affinity of individual antibody fragments for the targeted toxin, the characteristics of the venom and the amount of each toxin in the venom, which may undergo considerable variations within the same species from region to region [69].

Independently, the group of Pasteur Institute in Tunis has focused on alternative methods that consist of designing single-domain antibodies, also called VHH, derived from dromedary (Arabian camel) heavy-chain antibodies. VHH’s interest as research, diagnostic and therapeutic tools has rapidly emerged due to several potential advantageous properties over conventional antibodies [86]. VHH are easily expressed in recombinant bacteria with great stability and solubility. They are characterized by a high penetration into tissues due to their low MW (15 kDa) and cross the brain-barrier, and one usually considers that they are not immunogenic, because of their small size, rapid clearance from blood and high sequence homology with human VH. Because of these characteristics, the Tunisian group (Institut Pasteur, Tunis) first analyzed the serum of camels immunized with isolated scorpion neurotoxins and showed that heavy chain antibody subclass were capable of binding and neutralizing the toxins used for immunization [87]. Soon after, they constructed an immune VHH-phage library by following a well-established protocol previously described by others with the aim of isolating specific single-domain antibodies directed against the most potent toxins of Aa venom (AahI') [88]. Nanobody AahI'22 was selected and produced as a free monovalent, tandem-linked bivalent and bivalent chimeric nanobody fused to the human antibody Fc fragment. All of these molecules exhibited high affinity for their target with a K_D_ in the nanomolar range without any additional *in vitro* affinity maturation, as was required for anti-Cn antibody fragments derived from naive human libraries. Their neutralizing capacity was also evaluated after subcutaneous or intracerebral injection into mice of toxin AahI’ premixed with the antibody fragments. Varying amounts of toxin and antibody fragments were investigated; but, surprisingly, the lethality of mice injected with free toxin was not recorded, and control animals received only physiological water. In addition, no rescue assays were carried out in order to compare the protective capacity of the various antibody formats to each other [89]. In the same manner, Abderrazek *et al.* reported soon after the isolation of nanobodies directed against AahII, opening the door to the design of a bispecific molecule (NbF12-10) made of two nanobodies, similar in size to an scFv (25–30 kDa [84,90]. Bispecific NbF12-10 preserved high affinity for toxins belonging to each group and also protected mice experimentally envenomed with the pooled toxic fraction of Aah venom. When testing VHH’s potential, the most significant result reported was the protection of mice after sc injection of 1.5 LD_50_ venom, a slightly higher dose to 1 LD_100_ [84]. All mice survived after the injection of 85 µg of NbF12-10, even when observing a delay of 15 min between envenoming and treatment. This result was encouraging, but is lacking for more drastic conditions, including a higher amount of toxins and a lower amount of antibody fragments. It is likely that these conditions were dictated by the low protective capacity of anti-AahII VHH (NbAahII10) for which no protection test has been shown, whatever the variant is (natural or its improved version) [90,91]. More convincing results were reported and supported soon after by early preclinical studies [92].

## 7. Prospects and Concluding Remarks

Many interesting novel recombinant toxin-neutralizing antibody fragments are being developed, and recent research and pre-clinical outcome reports have confirmed their potential for clinical applications. Due to the relatively low complexity of scorpion venoms in terms of the number of medically important toxins, recombinant antibodies may represent an advantageous alternative to polyclonal antibody fragments if one does not take into account the cost of the industrial development of such molecules. One also must consider that similar problems were encountered with the early development of antibiotics that are now produced at a very low price worldwide. It must be underlined, as well, that the cost of treatments with recombinant antibodies can be significantly lowered, as is the case for the treatment of various cancers with Bevacizumab (Avastin), now in the range of 25–40 €/doses.

A limitation of recombinant antibodies is that one antibody will typically recognize only one of several toxin serotypes. Cutting edge approaches in development to address this shortcoming may consist of using cocktails of monoclonal antibodies, engineered antibodies to recognize multiple serotypes or even cocktails consisting of several unique antibodies, as reported by Symphogen for the treatment of cancer with improved efficacy [93,94].

**Figure 7:**
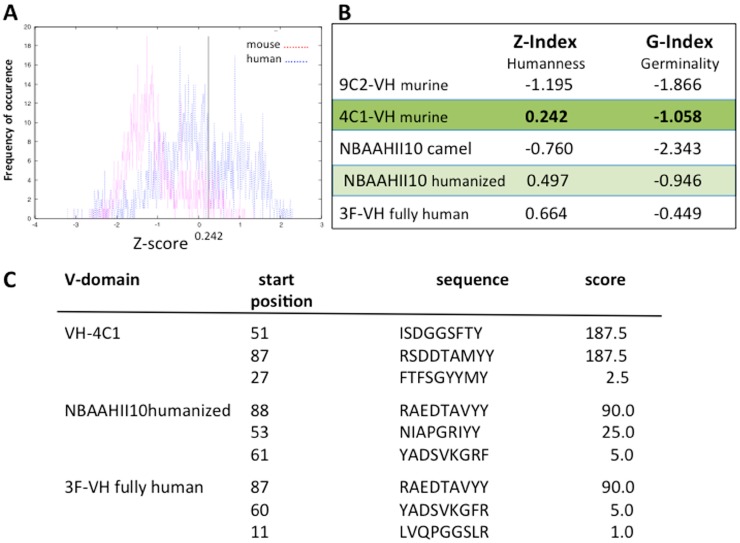
Humanness and germinality indexes of the V-domains of some antibody fragments directed against scorpion toxins. The antibody amino acid residue sequence is compared with a set of human sequences assigned or not to germline-derived families. The Z-score gives an overall similarity to circulating antibodies, while the G-score indicates the similarity to circulating antibodies derived from each human germline family. (**A**) Histogram of human and mouse pair-wise sequence identities in the 4C1-VH domain. The calculated Z-index is 0.242; (**B**) The Z-index and G-index for a set of V-domains; (**C**) Prediction and ranking of potential 9-mer peptides based on a predicted half-life of dissociation to HLA class I molecules (allele A1). Analysis was restricted to 4C1-VH, humanized NBAAHII10 and 3F-VH human variable domains.

Several strategies are now available to design recombinant neutralizing molecules. Engineering recombinant antibody fragments from monoclonal antibodies is rapid, efficient and leads to molecules that usually retain the antigen-binding characteristics of the parental antibody, even if one exception has been reported [95]. Fully-human antibody fragments have also been reported after panning naive libraries with interesting results, even if the strategy is laborious and requires, in all cases, reported *in vitro* affinity maturation. Finally, early preclinical analysis of single-domain antibodies selected from immune VHH-phage libraries has shown their neutralizing potential. One of these molecules was humanized in view of clinical applications, but one can wonder if the humanization of antibody variable domains is really required for the design of recombinant antivenom. Recent reports indicate that humanized antibody fragments are not always safer than chimeric human/mouse Fabs [96,97]. We should bear in mind that antivenoms are expected to be injected into the patient only once. Envenoming is not a chronic disease that requires repeated and long-term injection of antidote. When the humanness and germinality index of some anti-scorpion toxin antibodies were compared, we observed no significant differences between murine antibody (IGHV-4C1), humanized camelids (NBAAHII10) and fully-human IGHV-3F in terms of humanness (Z-Index) and germinality (G-Index) (Figure 7) [98]. HLA peptide binding prediction led to similar conclusions [99].

What is more important in preclinical evaluation is the assessment of the PK/PD properties and the capacity of novel antibody formats to inhibit toxins that have been injected into individuals previously and independently of the antivenom. It is essential not to confuse the efficacy of antivenom, defined as its ability to bind and neutralize venom-mediated effects under ideal conditions, and the effectiveness of antivenom, defined as its ability to reverse or prevent envenoming in human cases [100]. It is useless to claim that a particular fragment is more appropriate than another if its assessment was carried out only through neutralization assays consisting of the injection of premixed toxin and antibody to animals. Protective assays referring to the ability to inhibit lethal toxicity when the venom and the antidote are injected independently into animals via two distinct routes are more stringent tests that mimic the latency period required for the distribution of free toxins and antibodies in the body. This allows the venom components to distribute into the tissues according to their individual PK properties, which are still not well understood, despite extensive investigations [101]. Such assays should now be systematically standardized and carried out in comparison with conventional antivenoms. Neutralizing assays according to the WHO recommendations should be reserved for the development of fragments whose PK properties have yet to be well-characterized, as in the case for Fabs and F(ab')_2_.
